# Geopolymers: Advanced Materials in Medicine, Energy, Anticorrosion and Environmental Protection

**DOI:** 10.3390/ma16237416

**Published:** 2023-11-29

**Authors:** Sonia Kudłacik-Kramarczyk, Anna Drabczyk, Beata Figiela, Kinga Korniejenko

**Affiliations:** Department of Materials Engineering, Faculty of Materials Engineering and Physics, Cracow University of Technology, 37 Jana Pawła II Av., 31-864 Cracow, Poland; sonia.kudlacik-kramarczyk@pk.edu.pl (S.K.-K.); beata.figiela@pk.edu.pl (B.F.)

**Keywords:** geopolymers, geopolymer-based drug carriers, water purification, fire-resistant materials, thermal insulation, microbial protection, anticorrosive materials

## Abstract

The initial predictions of the importance of geopolymers primarily assumed use mainly in the construction sector. However, as research progresses, it is becoming clear that these versatile materials demonstrate the ability to greatly exceed their original applications, as characterized in detail in this review article. To the best of our knowledge, there is no literature review concerning geopolymer materials that compiles the diverse applications of these versatile materials. This paper focuses on geopolymer applications beyond the construction industry. The surprising application potential of geopolymers in medicine has become a topic of particular interest. Therefore, considerable attention in this paper is devoted to characterizing the utility of these materials in tissue engineering, dentistry and drug delivery systems. Geopolymers not only have exceptional heat resistance and compressive strength, making them durable and resistant to manipulation (over five times less drug released from the geopolymer carrier compared to the commercial formulation), but also provide a robust solution for extended-release drug delivery systems, especially in opioid formulations. Their chemical stability, porous structure and ability to maintain structure after repeated regeneration processes speak to their potential in water treatment. Geopolymers, which excel in the energy industry as refractory materials due to their resistance to high temperatures and refractory properties, also present potential in thermal insulation and energy storage. It was demonstrated that geopolymer-based systems may even be 35% cheaper than conventional ones and show 70% lower thermal conductivity. In terms of protection against microorganisms, the possibility of modifying geopolymers with antimicrobial additives shows their adaptability, maintaining their effectiveness even under high-temperature conditions. Research into their use as anticorrosion materials is targeting corrosion-resistant coatings, with geopolymers containing graphene oxide showing particularly promising results. The multitude of potential applications for geopolymers in a variety of fields reflects their enormous potential. As research progresses, the scope of their possibilities continues to expand, offering innovative solutions to pressing global challenges.

## 1. Introduction

Geopolymers are three-dimensionally structured materials formed as a result of chemical reactions between alkali silicates and silica precursors. This process, defined as geopolymerization, leads to the formation of durable and strong materials. Physicochemical properties of final geopolymers, including their workability, setting time and compressive strength, depend strongly on the type and properties of the reagents applied (precursors and activator) and the synthesis conditions (mixing and curing parameters) [1].

Over the past few years, geopolymers, initially envisioned mainly for applications in the construction sector, have become the subject of intense scientific research, demonstrating their capacity for a much wider range of applications than originally envisioned, as this review article explores in detail. These discoveries are revolutionizing the outlook on the potential of these versatile materials in various fields, which is becoming crucial in the face of today’s global challenges. Previous research on geopolymers seems to indicate their ability to transcend traditional areas of application, revealing the evolution of scientific thought on them. Although they were initially associated mainly with the construction sector, their potential in medicine has been observed and is becoming a special area of interest. A review of the literature in this area is important to gather and understand the variety of applications for geopolymers in the context of tissue engineering, dentistry and drug delivery systems [2,3].

The initial studies on geopolymers occurred during the 1950s and 1960s, with ongoing development in subsequent decades. Joseph Davidovits, a French chemist, played a pioneering role in geopolymer research by publishing papers on their structure and potential applications. Since then, the interest in geopolymers has steadily increased, leading to their progressively versatile utilization across various scientific and industrial fields [4,5].

In the past several years, there has been a focus on intensive research and exploration of geopolymers due to the unique properties of these materials and the consequent high potential for their applications in a variety of fields. One of the best-known areas of application for geopolymers is their use in the construction industry. For example, Nguyen et al. [6] investigated metakaolin-based geopolymers in terms of their potential application as walling in buildings. In their study, various fillers, including polystyrene and glass microspheres, were introduced into the geopolymer matrices to verify their impact on the properties of the final materials. It was demonstrated that the developed materials constituted an interesting alternative to the Portland cement used in the construction industry today [6]. In turn, in the work of Raza et al., the use of 3D-printed geopolymers in the construction industry has been widely discussed by showing both their advantages and disadvantages compared to conventional cements [7]. Asghar et al. clearly emphasized in their work the ecological aspect of the use of geopolymer-based concretes in the construction industry [8]. A similar discussion was performed by Ansari et al., who demonstrated that there is a clear need to look for materials that might replace conventionally applied cements for sustainable development [9]. Importantly, due to the fact that raw geopolymer materials such as fly ash or blast furnace slag are often byproducts of other industrial processes, their application significantly promotes more sustainable practices within the construction industry, both considering greenhouse gas emissions (up to an 80% reduction) and costs (up to a 30% reduction) [10]. Compared to conventional Portland cement, geopolymers offer numerous advantages, such as higher mechanical strength, greater environmental resistance and lower environmental impact [11,12]. Therefore, they are an attractive alternative to traditional construction materials [13,14].

However, the application area of geopolymers is not limited to construction. To the best of our knowledge, there is no literature review on geopolymers that presents the diverse applications of this group of materials. This paper shows their applications as drug carriers, dental implants and materials supporting bone regeneration; their use in removal of various chemical compounds from wastewater; as well as their potential in developing anticorrosive systems, systems showing antimicrobial activity and fire-resistant materials. Additionally, no review article presenting the application potential of geopolymers in biomedical areas was found; hence, in this review article, more attention was paid to exactly this aspect, which seems to be promising considering the fact that in the early stages, these materials were considered mainly for application in the construction industry. Little attention is paid to the application of geopolymers in this field since this area has been widely described in many papers.

This review skips the basic application of geopolymers in the construction industry, as this is well known from the literature, and explores new possibilities for the use of their advanced functions, including as a building material with special properties, that are very often marginalized. The article is based on a critical review of the literature, especially using search results from the Scopus database focused on keywords connected with geopolymers and applications, excluding the construction industry. This approach allows readers to discover a “new face” of geopolymer materials as an advanced multi-functional material dedicated to modern applications.

With advances in research, there is a growing recognition that geopolymers are not only a modern building material but also an advanced, versatile material that can significantly contribute to innovation in many areas of science and industry [15,16]. This review aims to demonstrate this evolving approach to geopolymer applications, departing from traditional perceptions of their role in the construction sector [17,18]. In addition, aspects related to the use of geopolymers in the fields of microbial protection, corrosion prevention or as refractory materials are being researched, directing attention to further promising areas for their application [19,20]. At the same time, existing research suggests that geopolymers can not only compete with but also outperform traditional materials in terms of economic and thermal efficiency [21,22].

## 2. Application of Geopolymers in Biomedical Areas

The application of geopolymers in medicine and other relevant areas is also being widely investigated nowadays. This is due to their excellent mechanical properties (including compressive strength and durability) and easy fabrication process [23,24]. Importantly, it is also possible to obtain geopolymer materials showing controlled porosity and high biocompatibility by applying adequate chemical compositions [25].

Material considered for biomedical purposes should show properties such as biocompatibility and bioactivity; thus, many investigations thus far have characterized geopolymers in terms of these properties. It has been widely demonstrated in many experiments that geopolymers demonstrate these properties as a result of the synthesis methodology applied or adequate modifying agents used during synthesis. For example, Pangdaeng et al. [26] proposed to treat a calcined kaolin-based geopolymer with calcium chloride to affect its physicochemical and biological properties. The obtained geopolymer samples were soaked in 0.5 M CaCl_2_ solution for 24 h at an ambient temperature. It was reported that the procedure applied resulted in better bioactivity of the geopolymers as well as in their higher compressive strength [26]. In turn, Ogun and Derun [27] performed studies on metakaolin-based geopolymers incorporated with bioactive inorganic compounds such as calcium phosphate and calcium hydroxide. The preliminary studies obtained materials showing high bioactivity in simulated body fluid (SBF). Firstly, the impact of the mentioned ceramics was verified, and it was concluded that the presence of both calcium compounds enhanced the mechanical properties of the developed materials [27]. The impact of the presence of calcium hydroxide within the geopolymer structure on its properties after soaking in an SBF solution was verified by Tippayasam et al. [28]. Here, it was clearly stated that the higher the calcium hydroxide content, the higher the bioactivity of the geopolymer manifested by the formation of a bigger apatite layer on its surface [28]. Preliminary studies preceding research aiming at verifying the bioactivity of geopolymer materials were also performed by Poolkwan et al. [29]. The first goal of the researchers was to obtain the material with the highest strength properties. It was reported that this was achieved in the case of a geopolymer doped with calcium phosphate, activated with NaOH and KOH and subjected to 600 °C [29].

Many investigations are currently being performed on the utility of geopolymers in fields such as stomatology, bone regeneration and drug delivery systems. Exemplary studies are described in more detail below.

### 2.1. Drug Delivery Systems

Geopolymers’ resistance to heat as well as their high compressive strength has resulted in the use of these materials during the preparation of tamper-resistant extended drug release systems. This is particular meaningful in the case of opioid release formulations. It is highly important to develop a formulation in which the active substance will be protected against pulverization and purposeful manipulation. Such investigations were performed by Cai et al. [30]. In their paper, a geopolymer paste based mainly on metakaolin was molded into cylindrical rods and pellets, and the prepared materials were defined as Formulation A. The notation Formulation B was applied to a commercial drug tablet—a tested oxycodone drug belonging to the opioids group. Based on the performed analyses, it was concluded that the geopolymer was a promising material for creating tamper-resistant formulations of active substances, particularly opioids. A comparison with a commercial formulation showed that the geopolymer was much more resistant to abuse attempts, requiring more time and effort to be tampered with. Additionally, the geopolymer enabled a controlled drug release, which may have an impact on reducing opioid abuse [30]. The geopolymer matrix as a vehicle for the controlled release of oxycodone was also investigated by Forsgren et al. [31]. Here, the dependence between the mesoporosity of the geopolymer and the drug release kinetics was determined [31].

Studies on the sustained release of an active substance from geopolymer-based formulations were also conducted by Jämstorp et al. [32]. The main purpose of the performed experiments was to determine the effect of the amount of water used during the synthesis of geopolymers on their porosity (pore structure and size) and thereby their ability to control the release of the drug. Moreover, the study also aimed to verify the effect of the compressive strength of the geopolymer material in terms of its usefulness as a drug carrier. It was reported that as the water content increased, the geopolymer porosity (and also the average pore size) and the drug release rate increased, while at the same time, the compressive strength of the tested materials decreased. Nonetheless, the compressive strength of the analyzed materials prevented the potential rapid release of the active substance caused by possible mechanical interactions [32]. In another work, the potential of geopolymer pellets of various sizes as drug vehicles was verified. It was reported that the solubility of active substances within the geopolymer matrix significantly affects its release profile from the vehicle [33].

### 2.2. Bone Regeneration

The interest in the potential of geopolymers in bone tissue regeneration has recently grown [25]. This is the reason why many investigations are currently being performed to verify the utility of these materials within this area. For example, Rahdi and Ahmad [34] performed studies on the development of porous metakaolin-based geopolymers as bone substitutes. During the experiments, the porosity of the materials obtained as well as their morphology were characterized. The most important studies included in vivo experiments performed using white male rabbits deriving from New Zealand. In the course of ongoing studies, it was demonstrated that metakaolin-based geopolymers showed biocompatibility and bioactivity. Moreover, the developed materials promoted dense trabecular bone formation within two weeks of implantation, increasing mature bone after four weeks with negligible inflammation [34].

In turn, in the work of de Andrade et al. [35], the use of geopolymers in the fabrication of porous composites as scaffolds for bone tissue regeneration was discussed. Here, metakaolin-based geopolymers incorporated with hydroxyapatite were investigated in terms of their porosity, compressive strength and crystallinity. Importantly, in vitro experiments were also conducted in the presence of human adipose-derived mesenchymal stem cells. The performed studies concluded that the geopolymer scaffold showed no negative impact on the tested cell line. Furthermore, cells formed a monolayer on its surface, which indicated the biocompatibility of the examined material and its non-cytotoxicity towards the tested cells [35].

Porosity is an important aspect of scaffolds designed for bone regeneration. It strongly affects both the cell attachment and further tissue formation growth [36]. Hence, Faza et al. [37] focused on the development of the component ratio—i.e., metakaolin (M) and an alkaline activator (AA) used during the geopolymerization process—providing the geopolymer scaffold with the most favorable porosity characteristics. The following ratios of M/AA were investigated: 1:1.0, 1:1.5, 1:2.0, 1:2.5 and 1:3.0. They demonstrated that the geopolymer obtained using M and AA with a ratio of 1:1.0 showed the most favorable porosity. The porosity of that geopolymer matrix was like the porosity of human spongy bone; thus, the developed material seemed to be very promising as a bone substitute [37].

Recent studies on geopolymers were directed toward discovering their potential in bone tissue regeneration. These materials appear promising, presenting the ability to promote bone tissue formation processes. The ability to adapt to different conditions, as well as the lack of toxicity to cells, make these materials capable of providing scaffolds for bone tissue regeneration.

### 2.3. Dental Implantology

The field of dental restorative materials also seems to be an area in which geopolymer materials may play a significant role. Studies on nanocomposites consisting of metakaolin-based geopolymers and carbonated apatite doped with strontium and magnesium designed for dentistry were performed by Sutanto et al. [38]. The mentioned dopants were used to promote bone regeneration. It was demonstrated in the research that the developed materials were characterized by favorable mechanical properties (including compressive strength, hardness and modulus elasticity) in terms of their potential dental applications. Importantly, none of the investigated materials showed cytotoxicity towards mouse embryonic fibroblasts, which was verified via a trypan blue test [38].

Studies on the application of geopolymers in the dental field were also conducted by Sunendar et al. [39]. In this work, metakaolin-based geopolymers incorporated with carbonate apatite doped with strontium and prepared in a chitosan solution were examined. In the course of the research, the chemical structure of the materials was characterized via Fourier transform infrared (FT-IR) spectroscopy (using Shimadzu 200-91538 spectrometer, Kyoto, Japan), wherein their mechanical properties (including hardness, flexural modulus and flexural strength) were evaluated using a universal testing machine (TENSILON UCT-5T, Tokyo, Japan). One of the main tasks of the performed studies was to check the impact of both strontium content and the amount of chitosan used during geopolymerization on the properties of the final materials. It was reported that the material containing carbonate apatite doped with strontium in the amount of 5% mol obtained using a 2% chitosan solution as a coupling agent showed the most favorable mechanical properties in terms of its potential dental implant application [39].

The next paper [40] discussed the impact of geopolymer–carbonate apatite nanocomposites on early bone healing. For this purpose, nanocomposite samples were introduced into the tibia of rabbits (New Zealand White male, 3.0–3.5 kg weight, age—6 months). On the 14th day of the study, reactive bone formation was reported. Moreover, osteoids, osteoblasts and osteocytes demonstrated higher maturity, and the woven bone was denser after 28 days [40].

Thus, to sum up, geopolymer materials appear promising for creating extended-release drug delivery systems for several key reasons. Their heat resistance and compressive strength make them more durable and more resistant to tampering than other materials. In the context of opioid formulation, this feature can significantly hinder drug abuse while increasing the safety of use. Additionally, the ability to release the active substance in a controlled manner makes geopolymers an ideal carrier for drugs, especially opioids. Compared to commercial formulations, geopolymers present significant resistance to manipulation, making them more reliable. All these factors make geopolymer materials a more attractive choice compared to other available materials, especially in the context of opioid drug safety.

In Table 1, the above-presented application of geopolymers for biomedical areas is briefly summarized.

## 3. Potential of Geopolymers in Water Purification

Geopolymer materials may be widely used as innovative absorbents for the removal of heavy metals [41], dyes [42], pharmaceutics [43], emulsified oil droplets [44] or surfactants [45] from wastewater. This is due to their chemical stability, porosity and thus large surface area. Maged et al. [46] mentioned several factors influencing the adsorption of pollutants by geopolymers. They indicated among others the porosity (the pore size) of geopolymers; their surface chemistry, including their hydrophilicity or hydrophobicity; as well as the presence of various functional groups on the surface of geopolymers affecting potential interactions between the adsorbent and the adsorbed substance [46]. The importance of the porosity in terms of the adsorption efficiency was also indicated by Tan et al. [47]. Here, the authors presented the relationship between the porosity and the specific surface area, which is a crucial factor in terms of effective adsorption [37]. Latorrata et al. [48] demonstrated that adsorption of metals within the geopolymer-based adsorbents involves the trapping of these substances into geopolymer pores [48].

Studies on the removal of heavy metals such as cadmium [49], lead [50,51], copper [52], chromium [53], nickel and cobalt [54] and iron and manganese [55] by geopolymer-based adsorbents have been performed.

Geopolymers have also been investigated in terms of the removal of ammonia from wastewater. Such studies were carried out, for instance, by Franchin et al. In their work, the fabrication of geopolymers via additive manufacturing was presented. The crucial aspect of their works was to verify the removal efficiency of ammonium from wastewater. It was reported that the developed materials showed a beneficial porosity, mechanical properties and high exchange capacity in terms of their potential application for wastewater purification. Importantly, it was also concluded that the geopolymers showed high removal efficiency and thus high potential for use as adsorbents [56]. Similar studies on the sequestration of ammonium ions and phosphate ions were described by Salam et al. [57]. Here, the effective purification of wastewater from the mentioned ions by developed zeolite/geopolymer composites was demonstrated [57].

The application potential of geopolymers in the purification of dyes from wastewater has also been widely discussed. The removal efficiency of geopolymer-based adsorbents was verified regarding various dyes, including acid green and procion red. For this purpose, Hua et al. [58] obtained a geopolymer material incorporated with magnetite to enable the separation of the adsorbent from the tested medium. The results of the performed experiments indicated that the developed adsorbent showed effectiveness in the removal of both mentioned dyes, and that the adsorption process did not depend on the temperature conditions [58]. Analysis of the adsorption of methylene blue by a fly-ash-based geopolymer material was performed by Alouani et al. [59]. This study determined the impact of parameters such as temperature, pH, dye concentration and contact time on the adsorption process. Research showed that the maximum adsorption efficiency was observed in the alkaline environment, wherein no significant effect of temperature conditions was observed [59]. Pimtaksa et al. [60] developed geopolymer–zeolite composites containing titanium dioxide and investigated the methylene blue adsorption capacity of these materials as well as their photocatalytic efficiency. It was reported that the dye adsorption capacity of composites doped with 40 wt.% TiO_2_ was 99.1%. Importantly, the composites also showed very good stability and high porosity [60]. Studies on the effectiveness of geopolymers in removing methylene blue from wastewater were also performed by Li et al. [61]. Here, physicochemical and adsorption properties of solid-waste-based geopolymer subjected to an acid treatment were analyzed. It was concluded that the developed materials demonstrated high porosity, excellent specific surface area and high removal efficiency, even after 10 regenerations [61]. The ability of geopolymer-based adsorbents to remove methylene blue from wastewater was also verified by Shikuku et al. [62], Papa et al. [63] and Novais et al. [64]. In the course of various studies, geopolymer materials have also been defined as effective adsorbents of dyes such as methyl orange dye [65], reactive red 195 dye [66], Rhodamine B and Congo red [67]. An approach based on the immobilization of organic dyes, including methylene blue, Congo red and acid blue by metakaolin-based geopolymers was investigated by Abdullah Al-Mashaqbeh et al. [67]. In their research, the geopolymerization process was performed in the presence of the mentioned organic dyes so as to immobilize them within the geopolymer matrix. Further studies involved the use of various leaching solutions (with various pH and ionic strengths) to verify the effectiveness of the immobilization [68]. Interesting approaches concerning the application of geopolymers in wastewater purification were presented by Luhar et al. [69]. In their paper, the application of geopolymers as pressure-driven membranes, filtration media and even pH-buffering agents was widely discussed [69].

Geopolymers are distinguishable by a number of desirable properties that make them promising for water purification. Their chemical stability and porosity make them effective adsorbents for various chemical compounds, including heavy metals, dyes and other pollutants. The large surface area available for interaction with pollutants enhances their effectiveness in adsorption processes. In addition, geopolymers exhibit the ability to release active substances in a controlled manner, which is crucial when treating wastewater for various pollutants. Their mechanical stability and ability to maintain their structure after repeated regeneration processes further emphasize their durability and cost effectiveness in long-term use. Combined with their potential for use as pressure-driven membranes and pH buffers, geopolymers have shown their versatility, making them a promising material in the field of water treatment.

## 4. Geopolymers in the Energy Industry

### 4.1. Role in the Development of Fire-Resistant Materials

Geopolymer materials demonstrate excellent heat resistance when exposed to elevated temperatures [70,71]. These materials possess inherent fire-resistant properties due to their inorganic structure, which distinguishes them from organic polymers. They do not combust, produce toxic substances or smoke when exposed to fire. Hence, they could be used in the fabrication of fire-resistant materials [72].

A study on the use of geopolymers for this purpose was performed by Galiano et al. [73]. In this work, investigations on the fabrication of fly-ash-based geopolymers for application as panels showing fire resistance were described. One of the analyzed variables was the type of alkaline activator used during the geopolymerization process. Hence, one series of geopolymers was obtained using sodium silicate, while the other used potassium silicate, and the studies aimed at verifying the impact of the type of activator used on the physicochemical properties of the final materials. It was demonstrated that regardless of the activator used, the geopolymers showed similar insulation characteristics in the panels. However, geopolymers obtained using sodium silicate exhibited better mechanical properties than samples prepared using potassium silicate. Importantly, all investigated geopolymer panels underwent the Dutch standard leaching test NEN 7345 [74], and it was demonstrated that the type of alkaline activator had no influence on the leachability of the tested panels [73]. In another work authored by Zhang et al. [75], mechanical investigations on geopolymers based on fly ash and metakaolin were presented. Experiments were performed on samples both at room temperature and after exposure to elevated temperature. The main objective of the research was to select an adequate proportion of fly ash and metakaolin to achieve the sample with the most favorable properties. It was demonstrated that the materials obtained with 50% fly ash and 50% metakaolin showed the optimum compressive and bending strength both at room temperature and after exposure to elevated temperatures (100, 300, 500, 700 and 800 °C). Moreover, it was concluded that the developed geopolymer materials showed mechanical properties comparable to the properties of Portland cement samples at all tested temperatures [75].

It has been reported in many studies that adequate modification of the geopolymer matrix may enhance its heat resistance. For example, it was demonstrated that the addition of chopped carbon fibers (2 wt.%) into a geopolymer matrix consisting of 50% metakaolin and 50% fly ash enhanced the bending properties of geopolymers previously exposed to high temperatures [76]. The enhancement of the fire resistance of geopolymer materials incorporated with fibers of various origins (including carbon, micro-steel and hooked steel) was also widely discussed by Alzeebaree et al. [77]. It has also been reported that the incorporation of select fillers, including alumina [78] and wollastonite [79], into geopolymers may also improve their fire resistance.

In turn, Sakkas et al. investigated the thermal, mechanical and physical properties of geopolymers based on SiO_2_-doped metakaolin, and the results obtained were compared to the results achieved for commercial materials showing fire resistance. The materials were exposed to fire, and the study was performed in line with the EFNARC guidelines for analysis of passive fire protection for materials such as tunnel linings. In the course of the performed research, it was demonstrated that the developed materials showed excellent fire resistance and so may be useful as effective fire-resistant barriers [80].

The composition of the reagents used during the geopolymerization process as well as their properties also affect the thermal properties of the final geopolymers. For example, Abbass et al. indicated that a high content of alumina in metakaolin enhanced the heat resistance of geopolymer materials. Moreover, it was also demonstrated that the higher the fineness of the metakaolin, the better the thermal properties of the geopolymer [81].

Le et al. [82] proved the impact of basalt fibers on the heat resistance of geopolymer-based materials. It was reported that the samples with a high content of these additives demonstrated very high mechanical stability, even after exposure to high temperatures. The flexural and compressive strength of basalt fiber-containing geopolymers showed the highest values at 1200 °C. It was stated that the developed materials demonstrated a slight change from an amorphous phase to a crystalline one after exposure to high temperatures, which resulted in improving their mechanical properties [82]. Similar conclusions were made by Aziz et al. [83], who reported that the compressive strength of the geopolymers increased after exposure to high temperature due to phase transformation and densification [83]. These changes in geopolymers’ properties caused by high temperatures make these materials more favorable for application in the construction industry than concrete-based materials [72,84].

**Figure 1:**
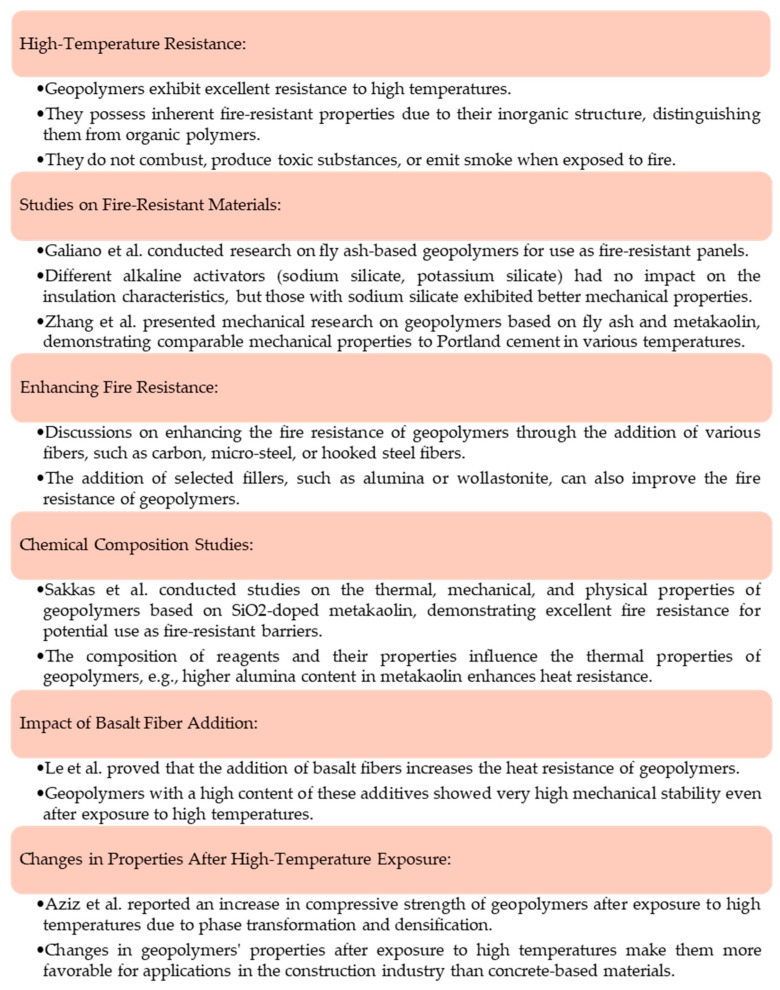
A schematic diagram describing the role of geopolymers in the fire-resistant materials industry [70,71,72,73,75,76,77,78,79,80,81,82,83,84].

Below, a scheme illustrating the significance of geopolymers in the fire-resistant materials industry is shown in Figure 1.

In summary, we can say that geopolymer materials possess exceptional heat resistance and inherent fire-resistant properties due to their inorganic structure, setting them apart from organic polymers. Various studies have explored their potential for fire-resistant applications, considering factors like alkaline activators and material modifications to enhance heat resistance.

### 4.2. Thermal Insulation

Thermal insulation may be defined as the reduction of thermal energy transfer between materials having different temperatures. It has been reported that thermal resistance reduces heat flow out of and into the material [85]. Due to their thermal properties, i.e., thermal conductivity and thermal diffusivity, geopolymer materials are widely discussed for thermal insulation applications, wherein particular attention is paid to geopolymer foams. For example, Zhang et al. [86] conducted studies on fly ash substituted partially with granulated blast furnace slag-based porous geopolymer materials. Their investigations verified the mechanical and thermal properties of geopolymers and determined their acoustic adsorption. It was reported that due to their relatively low thermal conductivity and diffusivity, the developed materials showed application potential for use as thermal insulation materials [86].

In another work, the potential of highly porous geopolymers (foams) for thermal insulation was investigated by Soe et al. [87]. Two series of porous geopolymers based on bottom ash were prepared using sodium lauryl ether sulfate (SLES) and aluminum powder as foaming agents. It was demonstrated that the materials obtained using SLES (5 wt.%) were characterized by high compressive strength and, importantly, approximately 70% lower thermal conductivity than a commercial clay brick. Hence, it was concluded that the developed geopolymers showed potential for application as thermal insulation construction materials [87]. An interesting concept was described by Ahmed et al. [88]. In their research, they used alumina waste and ferrosilicon slag to fabricate porous geopolymer bricks for thermal insulation. Hydrogen peroxide was applied as a foaming agent. The measured low conductivity of the fabricated materials indicated that they may be considered for thermal insulation applications. Moreover, it was demonstrated that the content of the foaming agent affected the microstructure of the geopolymers, which is important in terms of their thermal properties [88].

Bai et al. [89] fabricated metakaolin-based geopolymer materials (foams) via a gel casting method using hydrogen peroxide and Tween 80 as a foaming agent and surfactant, respectively. The low values of thermal conductivity for the obtained geopolymers, which were within the range of approximately 0.091 W/mK to 0.289 W/mK, indicated the application potential of the tested materials for thermal insulation [89]. Similarly low values for this parameter were reported by Bariş et al. [90]. Here, geopolymer materials obtained using volcanic tuff (an aluminosilicate source) and fine sand were also investigated in terms of their application for thermal insulation, and the results of the performed experiments confirmed their potential within this field [90]. In turn, Łach et al. [91] demonstrated that fly-ash-based geopolymer materials showing low thermal conductivity (>1.0 W/mK) were adequate for thermal insulation applications [91]. An interesting solution was proposed by Shahedan et al. [92]. Here, a glass bubble was employed as a fly ash replacement to improve the thermal and mechanical properties of geopolymers. It was reported that the higher the content of the glass bubble, the lower the values of thermal conductivity and thermal diffusivity of the tested materials. Hence, it was concluded that materials with glass bubbles showed high potential for thermal insulation applications [92]. In turn, Rashad et al. [93] analyzed geopolymers enhanced with expanded perlite and proved that this additive improved the thermal properties of the materials [93].

### 4.3. Further Possible Applications in the Energy Industry

An additional area in which the properties of geopolymer materials may be useful is their application potential for energy storage [23]. For example, Fang et al. [94] developed geopolymer aggregates as carriers of phase change (PC) materials. It was demonstrated that geopolymers may be incorporated even with approximately 16 wt.% of these materials. Importantly, the prepared system showed high mechanical strength and low thermal conductivity. Moreover, it was also reported that the materials obtained enabled thermal regulation; hence, they may be considered for the development of construction structures with energy-saving and temperature-regulating properties [94]. A similar application of geopolymers was also discussed by Zhang et al. [95]. Here, studies on hierarchically porous geopolymers based on kaolinite were presented, wherein the material was additionally modified with poly(ethylene glycol) (with a molecular weight of 4000 g/mol). The performed studies proved the high porosity and resulting high specific area of the developed PM carrier. Next, it was also concluded that the obtained materials exhibited excellent thermal properties, including low thermal conductivity as well as a high loading rate and high latent heat, which are particularly important in terms of energy storage applications [95].

The findings presented in other papers clearly indicate that geopolymers based on waste materials such as black slag and fly ash have shown excellent heat capacity, which is important in terms of their potential applications for thermal purposes. Furthermore, it was calculated that the developed systems are more cost-effective (up to 35% cheaper) than the two-tank systems based on molten salt applied so far [96]. In another article, it was established that geopolymers constitute adequate materials for encapsulation of selected molten chlorides; thus, phase change materials play an important role in thermal energy storage systems [97]. Yang et al. [98] proposed a geopolymer-based membrane forming a difunctional supercapacitor. This system also consisted of a pseudocapacitive electrode and an electrolyte (Na_2_SO_4_ solution). The porous structure of the geopolymer matrix enabled ion movement within the supercapacitor. It was demonstrated that the developed material exhibited high specific capacitance, thus showing a potential for application as a device for energy storage [98]. In another work [99], studies on geopolymer-based concrete were presented. As a result of the performed experiments, it was clearly reported that the thermal storage capacity (TSC) of this concrete can even be 3.5 times higher than the TSC of concretes based on ordinary Portland (OP) cements [99]. Rahjoo et al. also demonstrated that due to their thermal properties, including high thermal diffusivity and thermal energy storage capacity, geopolymer-based concretes may be an interesting alternative to OP cement-based concretes [100].

**Figure 2:**
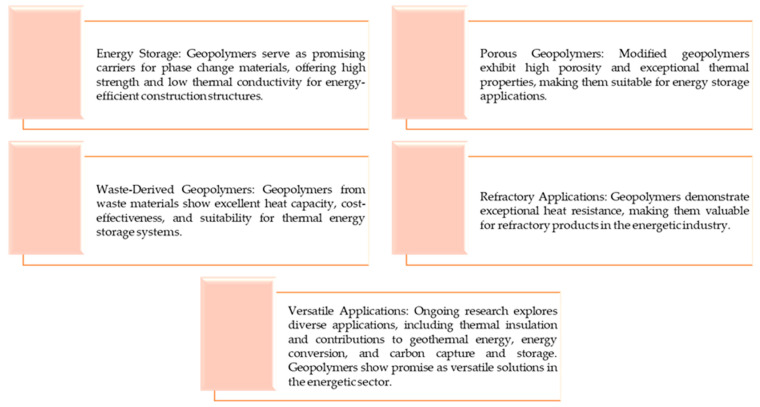
A schematic diagram describing the role of geopolymers in the energy industry.

Below, a summary scheme presents the conclusions regarding the application of geopolymers in the energy industry (Figure 2).

Currently, many studies are being performed to determine the potential of geopolymers in the energy industry, including their usability in geothermal energy applications [101], energy conversion [102] and carbon capture and storage [103].

To conclude, geopolymers in the energy industry show exceptional heat resistance, making them a promising material for developing refractory products. Due to their inorganic structure, geopolymers are fire resistant, emitting no toxic substances or smoke when exposed to fire. Studies on the use of geopolymers in the production of refractory materials have confirmed their effectiveness. Modifications to the geopolymer matrix, such as the addition of carbon fibers or other fillers to increase the mechanical properties and high-temperature strength, have also been widely discussed. Geopolymers, due to their thermal properties, are also being considered as effective materials for thermal insulation, especially in the form of foams. Research into their use as thermal insulation materials confirms their potential to reduce thermal conductivity, making them an attractive choice in the energy industry. In addition, the potential of geopolymers as energy storage materials is also being explored, opening up new prospects for their use in the energy field.

## 5. Geopolymer Applications in Microbial Protection

In recent years, the potential application of geopolymer materials for fabrication of systems showing antimicrobial properties has been observed [104,105]. The antimicrobial activity of geopolymer-based materials was investigated by Rondinella et al. Here, a metakaolin-based geopolymer was used to form a multilayer coating on a prosthetic device made of a Ti_6_Al_4_V alloy. The developed coating showed antibacterial activity by limiting bacterial growth [106]. In turn, Hashimoto et al. [107] verified the antimicrobial activity of metakaolin-based geopolymers immersed for 24 h in a copper chloride solution. The performed studies confirmed the antifungal properties of the obtained materials, which limited the growth of oyster mushrooms in a tested medium [107].

Importantly, Růžek et al. [108] clearly indicated that incorporation of various additives like inorganic metal particles (nano- or microparticles), inorganic metal ions or selected organic agents may enhance a geopolymer-based coating with antimicrobial activity [98]. For example, Armayani and Pratama [109] developed a geopolymer composite incorporated with silver nanoparticles. In the course of ongoing research, they demonstrated that the obtained material restrained bacterial growth and, importantly, also showed high heat resistance [109]. Nanosilver as a component of geopolymers showing antibacterial activity was also employed by Lira et al. [110]. During their research, geopolymer spheres based on calcined waste pen shells and coal fly ash were prepared and subsequently coated with amoxicillin and silver nanoparticles. It was demonstrated that the developed materials showed strong antibacterial properties towards *Escherichia coli* [110]. In turn, geopolymers based on fly ash and calcined shells and additionally coated with silver nanoparticles were investigated by Cerna et al. [111]. Research presented in this work also confirmed the antibacterial activity of the developed materials towards *E. coli* [111]. Gutiérrez et al. [102] presented geopolymer materials (mortars) containing such additives as copper oxide and titanium oxide nanoparticles as well as glass waste. Here, the antibacterial properties of the obtained geopolymer mortars against bacterial strains such as *E. coli*, *P. aeruginosa* and *S. aureus* were demonstrated [112].

Geopolymers, gaining recognition in the context of protection against microorganisms, exhibit unique properties that increase their attractiveness compared to other materials. Their ability to be modified through additives, such as silver nanoparticles or other antimicrobial substances, allows geopolymers to be tailored to specific needs. They also show the ability to maintain antimicrobial properties under high temperature conditions, making them promising in areas where both protection from microorganisms and thermal resistance are crucial.

## 6. Geopolymers as Anticorrosive Solutions in Industry

Over the past few years, there has been a surge of investigations dedicated to exploring the potential of geopolymers in the development of corrosion-resistant materials. For example, Zhang et al. [113] performed studies on metakaolin-based geopolymers incorporated with granulated blast furnace slag to determine their potential as anticorrosive coatings for concrete protection in the marine environment. They verified several parameters of the developed materials, including their permeability, setting times and the volume stability. It was reported that the geopolymer coatings showed very good anticorrosive properties. Importantly, it was also concluded that the average bond strength between the cement paste and geopolymer coating was over 1.5 MPa [113]. Jiang et al. [114] clearly emphasized the high application potential of geopolymer coatings. This is due to the numerous advantages of these materials, including their life cycle sustainability, environmental compatibility and long-term performance [114]. In turn, Aguirre-Guerrero et al. [115] investigated the effectiveness of coatings based on geopolymers obtained using metakaolin and alkaline-activated fly ash. Here, it was demonstrated that the developed coating materials showed excellent anticorrosive properties, thus prolonging the functionality of such coated concretes in marine environments [115]. In the research by Zhang et al. [116], it was concluded that the microstructure of developed geopolymer coatings significantly reduced the penetration of seawater within concretes with such coatings [116]. In turn, Yang et al. [117] obtained geopolymer coatings containing reduced graphene oxide (rGO) and tested developed systems in terms of their anticorrosive properties using a 3.5% solution of NaCl. It was demonstrated that rGO-containing geopolymer materials showed increased corrosion resistance in the tested medium compared to the resistance of bare carbon steel [117]. In another work presented by Tomar et al. [118], studies on geopolymers based on fly ash and red mud were described. Next, the spray technique was applied to coat mild steel using the developed geopolymers. The experimental part included various investigations, with the most important one verifying the corrosion resistance of the geopolymer-coated steel by means of the accelerated salt fog test performed in line with appropriate standards. Based on the performed tests, it was concluded that the geopolymer-based coating provided excellent anticorrosive properties [118]. An analysis of geopolymer-based coatings in terms of their corrosion resistance was also carried out by Gupta et al. [119]. During these investigations, class F fly-ash-based geopolymer coatings were subjected to static salt (3.5% NaCl solution) and water immersion tests for 90 days. Importantly, the weight of the coatings as well as their adhesion strength were investigated both before and after immersion (every 15 days). Additionally, an accelerated salt test was also conducted. The performed studies confirmed that the formulated coatings upheld the point load (up to 5 kg) and demonstrated appropriate salt and water resistance (up to 3 months) [119].

Tomar et al. [120] conducted research on the application of geopolymer materials for corrosion protection. They developed innovative anticorrosive microcoatings based on a geopolymer with the addition of TiO_2_, aiming to effectively inhibit the oxidation of low-carbon steel in challenging environmental conditions. The studies suggest that these microcoatings can serve as effective corrosion protection in aggressive environments. Zainal et al. [121] investigated the use of geopolymers for corrosion protection, confirming the effectiveness of geopolymers in forming durable protective layers on the surface of carbon steel. Morphological analysis, open circuit potential (OCP) and adhesive strength tests indicated that the geopolymer anticorrosive layer develops over time, improving its properties. This suggests that geopolymers could be a promising material in corrosion protection, offering durability and effective protection for steel in corrosive conditions. Additionally, Morla et al. [122] conducted research on the corrosion resistance of fly ash and bottom ash-based geopolymer concrete. The results demonstrated that geopolymer concrete produced using these ash materials exhibited anticorrosive properties. These studies suggested that geopolymer concrete could be an effective alternative in terms of corrosion resistance and potentially significant for the durability of building structures. In their article [123], Omel et al. proved that the use of geopolymers in concrete pipes significantly improved the corrosion resistance of these structures in aggressive environments due to lower concentrations of corroding substances, higher density and better material hardening compared to traditional cement. Furthermore, experiments showed that geopolymers have significant benefits in terms of corrosion resistance compared to Portland-cement-based concrete, making them a promising material for water and sewage infrastructure applications. Additionally, in their article [124], Sobhan et al. demonstrated that fly-ash-based geopolymer structural concrete (GPC), with or without fibers, exhibits corrosion resistance in a marine environment. The study involved using an electrochemical method to induce accelerated corrosion. Samples of a beam with polyethylene fibers in amounts of 0.1%, 0.3% and 0.5% by volume were subjected to artificial corrosion, allowing for a faster and measurable assessment of corrosion resistance. As a result of the analysis, fiber-reinforced GPC beams (0.1–0.5%) showed a 24% reduction in crack scores and a 109% increase in residual flexural load capacity compared to unreinforced corroded GPC beams. These results indicate the promising application of fiber-reinforced geopolymer concrete as a durable structural material in a marine environment [124].

Thus, to summarize, studies on the use of geopolymers as anticorrosion materials are attracting attention because of their potential in this field. Recent work has focused on developing effective anticorrosion coatings based on geopolymers. For example, research on geopolymers containing graphene oxide (rGO) has shown improved corrosion resistance compared to traditional steels in a brine environment. Other experiments have confirmed the excellent durability of geopolymer coatings on metal, suggesting their promising application in corrosion protection. Therefore, it can be concluded that the performed experiments open up new possibilities for future applications of geopolymers in the field of anticorrosion material technology.

## 7. Summary

### 7.1. Conclusions

- Geopolymer materials, initially investigated mainly for the construction industry, have shown wide application potential in fields such as medicine, environmental protection and the energy industry. Importantly, they play an important role in the design of materials with antimicrobial and anticorrosive properties.
- Geopolymers are gaining attention in medicine due to their excellent mechanical properties, controlled porosity and biocompatibility. In the field of drug delivery, their strength allows for the formation of safe release systems for active substances, especially opioids. In bone regeneration and dental implantology, geopolymers show the ability to promote bone tissue formation.
- Geopolymers may be applicable for the removal of a variety of contaminants, including heavy metals, dyes, pharmaceuticals and surfactants from wastewater. Their chemical stability, porosity and large surface area make these materials a versatile tool for effective removal of contaminants through adsorption processes, making them a promising material in the field of water purification.
- Geopolymers show excellent resistance to high temperatures, which makes them promising for the fabrication of fire-resistant materials. Improving the high-temperature resistance of geopolymers, while improving their mechanical properties, may be achieved by introducing modifiers such as carbon fibers into the geopolymer matrix. Geopolymers, due to their thermal properties, are also being considered as effective materials for thermal insulation. Research into their use as insulation materials confirms their potential to reduce thermal conductivity, making them an attractive choice in the energy industry. Moreover, studies on the potential of geopolymers as energy storage materials are opening up new prospects for their use in the energy field.
- Geopolymers are gaining recognition in terms of protection against microorganisms. The possibility of incorporating additives such as silver nanoparticles or other antimicrobial substances into the geopolymer matrix allows geopolymers to be tailored to specific applications. In addition, they demonstrate the capability of maintaining antimicrobial activity even at high temperatures, which makes them useful in fields where protection against various microorganisms while maintaining heat resilience is critical.
- Geopolymers, as anticorrosion materials, are gaining the attention of researchers. Current studies are focusing on developing effective anticorrosion coatings based on geopolymers. Moreover, other experiments have confirmed the excellent durability of geopolymer coatings on metal, suggesting their promising application in corrosion protection.

### 7.2. Future Directions and Limitations

The evolution of geopolymers, from building materials to versatile tools for many industries, including medicine and pharmacology, reflects their transformative potential. As research advances, the scope of their capabilities continues to expand, offering innovative solutions to pressing global challenges. In short, discovering the undeveloped potential of geopolymers is a promising prospect for both the scientific and industrial communities.

Looking ahead, several key areas deserve attention for further research and development:

- Sustainable Geopolymer Production:

Efforts should be directed toward increasing the sustainability of geopolymer production processes. Investigating alternative raw materials, optimizing energy consumption and exploring eco-friendly curing methods can contribute to the overall environmental friendliness of geopolymers, in line with the growing demand for sustainable practices in various industries.

- Adapting Geopolymers to Specific Applications:

Customizing geopolymers for specific applications through additives or modifiers is a fascinating field. Fine-tuning the composition of geopolymers to solve unique challenges in a variety of fields, such as drug delivery systems, specialized water purification processes or application-specific anticorrosion coatings, can open up new possibilities for their use.

- Advances in Nanotechnology:

Further research into nanotechnology within geopolymers could yield breakthrough results. The introduction of advanced nanoparticles, nanotubes or nanofibers could enhance the mechanical, thermal and antimicrobial properties of geopolymers, pushing the limits of their performance in various applications.

- Biomedical Applications and Drug Delivery Systems:

In the biomedical field, there is a need for in-depth research focusing on the long-term biocompatibility and safety of geopolymers. Investigating their potential in sustained-release drug delivery systems, tissue engineering and other biomedical applications will be key to expanding their role in improving medical technologies.

- Standardization and Regulation:

As geopolymers find applications in various sectors, it will be important to establish standardized testing and regulation methods. This will ensure that geopolymers are consistent and reliable across applications, strengthening confidence among researchers, industrialists and regulators.

- Life Cycle Assessment:

Conducting a comprehensive life cycle assessment of geopolymers can provide information on their overall environmental impact. Understanding factors such as recyclability, durability and end-of-life issues will be key to establishing geopolymers as sustainable alternatives in the long run.

Despite the tremendous future potential for geopolymers, it is important to consider their potential limitations and challenges. These may include issues related to scalability, cost effectiveness and the need for widespread acceptance across industries. Confronting these challenges will be key to uncovering the full potential of geopolymers and ensuring their seamless integration into a variety of applications.

## Figures and Tables

**Table 1:** Table summarizing the application of geopolymer materials in biomedical areas [25,30,31,32,33,34,35,36,37,38,39,40].

Application of Geopolymers in Biomedical Areas		
Drug delivery systems	Excellent mechanical properties	Geopolymers show promise in extended drug release systems, especially for opioids, due to heat resistance and high compressive strength. Geopolymers are more resistant to tampering than traditional drug formulations, requiring increased effort for manipulation. Geopolymers facilitate controlled drug release, potentially reducing opioid abuse. Experiments on the geopolymer matrix reveal a correlation between mesoporosity and drug release kinetics. Geopolymers’ effectiveness as drug carriers depends on optimal component ratios for porosity and compressive strength.
	Controlled drug release	
Bone regeneration	Development of porous geopolymers as bone substitutes	Metakaolin geopolymers are biocompatible, promoting dense trabecular bone formation. Geopolymers with hydroxyapatite were tested for safety, showing favorable properties for mesenchymal stem cells. Porosity studies of bone regeneration scaffolds focused on optimal component ratios (M/AA) during geopolymerization. An M/AA ratio of 1:1.0 generates a porous geopolymer matrix resembling human cancellous bone, indicating potential as a tissue regeneration substitute.
	Biocompatibility and bioactivity	
Dental implantology	Use of geopolymers in dental restorative materials	Study on metakaolin geopolymers promoting non-cytotoxic bone regeneration. Investigation of metakaolin geopolymers with optimal mechanical properties for dental implants. Study on geopolymer–carbonate apatite nanocomposites and early bone healing. Geopolymers promising in extended drug release systems, particularly in opioid safety, due to resistance to manipulation and controlled release of active substances.
	Mechanical properties and biocompatibility	

## Data Availability

The data that support the findings of this study are contained within the article.

## References

[1] Castillo H., Collado H., Droguett T., Vesely F., Garrido P., Palma S. (2022). State of the art of geopolymers: A review. e-Polymers.

[2] Wu Y., Lu B., Bai T., Wang H., Du F., Zhang Y., Cai L., Jiang C., Wang W. (2019). Geopolymer, green alkali activated cementitious material: Synthesis, applications, and challenges. Constr. Build. Mater..

[3] Shehata N., Sayed E.T., Ali Abdelkareem M. (2021). Recent progress in environmentally friendly geopolymers: A review. Sci. Total Environ..

[4] Davidovits J. (2017). Geopolymers: Ceramic-Like Inorganic Polymers. J. Ceram. Sci. Technol..

[5] Komnitsas K., Zaharaki D. (2007). Geopolymerisation: A review and prospects for the minerals industry. Miner. Eng..

[6] Nguyen V.V., Le V.S., Louda P., Szczypiński M.M., Ercoli R., Růžek V., Łoś P., Prałat K., Plaskota P., Pacyniak T. (2022). Low-Density Geopolymer Composites for the Construction Industry. Polymers.

[7] Raza M.H., Zhong R.Y. (2022). A sustainable roadmap for additive manufacturing using geopolymers in construction industry. Resour. Conserv. Recycl..

[8] Asghar R., Khan M.A., Alyousef R., Javed M.F., Ali M. (2023). Promoting the green Construction: Scientometric review on the mechanical and structural performance of geopolymer concrete. Constr. Build. Mater..

[9] Ansari M.A., Shariq M., Mahdi F. (2023). Geopolymer concrete for clean and sustainable construction—A state-of-the-art review on the mix design approaches. Structures.

[10] Nawaz M., Heitor A., Sivakumar M. (2020). Geopolymers in construction-recent developments. Constr. Build. Mater..

[11] Li Z., Zhang J., Wang M. (2020). Structure, Reactivity, and Mechanical Properties of Sustainable Geopolymer Material: A Reactive Molecular Dynamics Study. Front. Mater..

[12] Matsimbe J., Dinka M., Olukanni D., Musonda I. (2022). Geopolymer: A Systematic Review of Methodologies. Materials.

[13] Lingyu T., Dongpo H., Jianing Z., Hongguang W. (2021). Durability of geopolymers and geopolymer concretes: A review. Rev. Adv. Mat. Sci..

[14] Petermann J.C., Saeed A., Hammons M.I. (2010). Alkali-Activated Geopolymers: A Literature Review.

[15] Castillo H., Collado H., Droguett T., Sánchez S., Vesely M., Garrido P., Palma S. (2021). Factors affecting the compressive strength of geopolymers: A review. Minerals.

[16] Bai C., Colombo P. (2018). Processing, properties and applications of highly porous geopolymers: A review. Ceram. Int..

[17] Alhawat M., Ashour A., Yildirim G., Aldemir A., Sahmaran M. (2022). Properties of geopolymers sourced from construction and demolition waste: A review. J. Build. Eng..

[18] Kumar A.S., Muthukannan M., Krishna I.S. (2020). Optimisation of bio medical waste ash in GGBS based of geopolymer concrete. Mater. Sci. Eng..

[19] Liu C., Huang X., Wu Y.Y., Deng X., Liu J., Zheng Z., Hui D. (2020). Review on the research progress of cement-based and geopolymer materials modified by graphene and graphene oxide. Nanotechnol. Rev..

[20] Mohd Mortar N.A., Abdullah M.M.A.B., Abdul Razak R., Abd Rahim S.Z., Aziz I.H., Nabiałek M., Ghazali M.F. (2022). Geopolymer ceramic application: A review on mix design, properties and reinforcement enhancement. Materials.

[21] Suresh Kumar A., Muthukannan M., Arun Kumar K., Chithambar Ganesh A., Kanniga Devi R. (2022). Development of environmental-friendly geopolymer concrete using incinerated biomedical waste ash. Recent Advances in Structural Engineering and Construction Management: Select Proceedings of ICSMC 2021.

[22] Wang Y.S., Alrefaei Y., Dai J.G. (2019). Silico-aluminophosphate and alkali-aluminosilicate geopolymers: A comparative review. Front. Mater..

[23] Cong P., Cheng Y. (2021). Advances in geopolymer materials: A comprehensive review. J. Traffic Transp. Eng..

[24] Elgarahy A.M., Maged A., Eloffy M.G., Zahran M., Kharbish S., Elwakeel K.Z., Bhatnagar A. (2023). Geopolymers as sustainable eco-friendly materials: Classification, synthesis routes, and applications in wastewater treatment. Sep. Purif. Technol..

[25] Ricciotti L., Apicella A., Perrotta V., Aversa R. (2023). Geopolymer Materials for Bone Tissue Applications: Recent Advances and Future Perspectives. Polymers.

[26] Pangdaeng S., Sata V., Aguiar J.B., Pacheco-Torgal F., Chindaprasirt J., Chindaprasirt P. (2016). Bioactivity enhancement of calcined kaolin geopolymer with CaCl_2_ treatment. Sci. Asia.

[27] Ogun B.S., Derun E.M. (2019). Investigation of nanoparticle use in geopolymer production. Eurasian J. Biol. Chem. Sci..

[28] Tippayasam C., Sutikulsombat S., Kamseu E., Rosa R., Thavorniti P., Chindaprasirt P., Leonelli C., Heness G., Chaysuwan D. (2019). In vitro surface reaction in SBF of a non-crystalline aluminosilicate(geopolymer) material. J. Aust. Ceram. Soc..

[29] Poolkwan K., Asavapisit S., Piyapanuwat R. (2022). Properties of hydroxyapatite-based geopolymer synthesized from calcined kaolin. IOP Conference Series: Materials Science and Engineering.

[30] Cai B., Engqvist H., Bredenberg S. (2014). Evaluation of the resistance of a geopolymer-based drug delivery system to tampering. Int. J. Pharm..

[31] Forsgren J., Pedersen C., Strømme M., Engqvist H. (2011). Synthetic Geopolymers for Controlled Delivery of Oxycodone: Adjustable and Nanostructured Porosity Enables Tunable and Sustained Drug Release. PLoS ONE.

[32] Jämstorp E., Strømme M., Frenning G. (2011). Modeling structure–function relationships for diffusive drug transport in inert porous geopolymer matrices. J. Pharm. Sci..

[33] Jämstorp E., Strømme M., Bredenberg S. (2012). Influence of Drug Distribution and Solubility on Release from Geopolymer Pellets—A Finite element Method Study. J. Pharm. Sci..

[34] Radhi H.A.A., Ahmad M.A. (2023). Biological Test of Porous Geopolymer as a Bone Substitute. J. Med. Chem. Sci..

[35] de Andrade R., Paim T.C., Bertaco I., Naasani L.S., Buchner S., Kovářík T., Hájek J., Wink M.R. (2023). Hierarchically porous bioceramics based on geopolymer-hydroxyapatite composite as a novel biomaterial: Structure, mechanical properties, and biocompatibility evaluation. Appl. Mater. Today.

[36] Murphy C.M., O’Brien F.J. (2010). Understanding the effect of mean pore size on cell activity in collagen-glycosaminoglycan scaffolds. Cell Adh. Migr..

[37] Faza Y., Harmaji A., Takarini V., Hasratiningsih Z., Cahyanto A. (2019). Synthesis of Porous Metakaolin Geopolymer as Bone Substitute Materials. Key Eng. Mater..

[38] Sutanto D., Satari M.H., Hernowo B.S., Priosoeryanto B.P., Septawendar R., Asri L.A.T.W., Purwasasmita B.S. (2020). Geopolymer–carbonated apatite nanocomposites with magnesium and strontium trace elements for dental restorative materials. J. Korean Ceram. Soc..

[39] Sunendar B., Fathina A., Harmaji A., Mardhian D.F., Asri L., Widodo H.B. (2017). The effect of CHA-doped Sr addition to the mechanical strength of metakaolin dental implant geopolymer composite. AIP Conference Proceedings.

[40] Sutanto D., Satari M.H., Hernowo B.S., Priosoeryanto B.P., Septawendar R., Asri L.A.T.A., Purwasasmita B.S. (2021). In vivo histomorphological evaluation of geopolymer-carbonated apatite nanocomposites implanted on rabbit tibia at early bone healing. Padjadjaran J. Dent..

[41] Grba N., Baldermann A., Dietzel M. (2023). Novel green technology for wastewater treatment: Geo-material/geopolymer applications for heavy metal removal from aquatic media. Int. J. Sediment Res..

[42] Maleki A., Mohammad M., Emdadi Z., Asim N., Azizi M., Safaei J. (2020). Adsorbent materials based on a geopolymer paste for dye removal from aqueous solutions. Arab. J. Chem..

[43] Wang X., Zhang Z., Ge Y. (2022). Oleic acid-tailored geopolymer microspheres with tunable porous structure for enhanced removal from tetracycline in saline water. Sustainability.

[44] Song Y., Li Z., Zhang J., Tang Y., Ge Y., Cui X. (2020). A Low-Cost Biomimetic Heterostructured Multilayer Membrane with Geopolymer Microparticles for Broad-Spectrum Water Purification. ACS Appl. Mater. Interfaces.

[45] Siyal A.A., Shamsuddin M.R., Rabat N.E., Zulfiqar M., Man Z., Low A. (2019). Fly ash based geopolymer for the adsorption of anionic surfactant from aqueous solution. J. Clean. Prod..

[46] Maged A., El-Fattah H.A., Kamel R.M., Kharbish S., Elgarahy A.M. (2023). A comprehensive review on sustainable clay-based geopolymers for wastewater treatment: Circular economy and future outlook. Environ. Monit. Assess..

[47] Tan T.H., Mo K.H., Ling T.C., Lai S.H. (2020). Current development of geopolymer as alternative adsorbent for heavy metal removal. Environ. Technol. Innov..

[48] Latorrata S., Balzarotti R., Adami M.I., Marino B., Mostoni S., Scotti R., Bellotto M., Cristiani C. (2021). Wastewater Treatment Using Alkali-Activated-Based Sorbents Produced from Blast Furnace Slag. Appl. Sci..

[49] Lan T., Guo S., Li X., Guo J., Bai T., Zhao Q., Yang W., Li P. (2020). Mixed precursor geopolymer synthesis for removal of Pb(II) and Cd(II). Mater. Lett..

[50] Wei E., Wang K., Muhammad Y., Chen S., Dong D., Wei Y., Fujita T. (2022). Preparation and conversion mechanism of different geopolymer-based zeolite microspheres and their adsorption properties for Pb^2+^. Sep. Purif. Technol..

[51] Novais R.M., Buruberri L.H., Seabra M.P., Labrincha J.A. (2016). Novel porous fly-ash containing geopolymer monoliths for lead adsorption from wastewaters. J. Hazard. Mater..

[52] Yu Z., Song W., Li J., Li Q. (2020). Improved simultaneous adsorption of Cu(II) and Cr(VI) of organic modified metakaolin-based geopolymer. Arab. J. Chem..

[53] He P.Y., Zhang Y.J., Chen H., Han Z.C., Liu L.C. (2020). Low-cost and facile synthesis of geopolymer-zeolite composite membrane for chromium (VI) separation from aqueous solution. J. Hazard. Mater..

[54] Ghani U., Hussain S., Noor-ul-Amin, Imtiaz M., Khan S.A. (2020). Laterite clay-based geopolymer as a potential adsorbent for the heavy metals removal from aqueous solutions. J. Saudi Chem. Soc..

[55] Pachana P.K., Rattanasak U., Nuithitikul K., Jitsangiam P., Chindaprasirt P. (2022). Sustainable utilization of water treatment residue as a porous geopolymer for iron removal from groundwater. J. Environ. Manag..

[56] Franchin G., Pesonen J., Luukkonen T., Bai C., Scanferla P., Botti R., Carturan S., Innocentini M., Colombo P. (2020). Removal of ammonium from wastewater with geopolymer sorbents fabricated via additive manufacturing. Mater. Des..

[57] Salam M.A., Mokhtar M., Albukhari S.M., Baamer D.F., Palmisano L., AlHammadi A.A., Abukhadra M.R. (2021). Synthesis of zeolite/geopolymer composite for enhanced sequestration of phosphate (PO_4_^3−^) and ammonium (NH_4_^+^) ions; equilibrium properties and realistic study. J. Environ. Manag..

[58] Hua P., Sellaoui L., Franco D., Netto M.S., Dotto G., Bajahzar A., Belmabrouk H., Bonilla-Petriciolet A., Li Z. (2020). Adsorption of acid green and procion red on a magnetic geopolymer based adsorbent: Experiments, characterization and theoretical treatment. Chem. Eng. J..

[59] El Alouani M., Alehyen S., EL Achouri M., Taibi M. (2018). Removal of Cationic Dye-Methylene Blue-from Aqueous Solution by Adsorption on Fly Ash-based Geopolymer. J. Mater. Environ. Sci..

[60] Pimraksa K., Setthaya N., Thala M., Chindaprasirt P., Murayama M. (2020). Geopolymer/Zeolite composite materials with adsorptive and photocatalytic properties for dye removal. PLoS ONE.

[61] Li C.J., Zhang Y.J., Chen H., He P.Y., Meng Q. (2022). Development of porous and reusable geopolymer adsorbents for dye wastewater treatment. J. Clean. Prod..

[62] Shikuku V.O., Tome S., Hermann D.T., Tompsett G.A., Timko M.T. (2022). Rapid Adsorption of Cationic Methylene Blue Dye onto Volcanic Ash-metakaolin Based Geopolymers. Silicon.

[63] Papa E., Mor M., Natali Murri A., Landi E., Medri V. (2020). Ice-templated geopolymer beads for dye removal. J. Colloid Interface Sci..

[64] Novais R.M., Ascensão G., Tobaldi D.M., Seabra M.P., Labrincha J.A. (2018). Biomass fly ash geopolymer monoliths for effective methylene blue removal from wastewaters. J. Clean. Prod..

[65] Purbasari A., Ariyanti D., Fitriani E. (2023). Adsorption of Methyl Orange Dye by Modified Fly Ash-Based Geopolymer–Characterization, Performance, Kinetics and Isotherm Studies. J. Ecol. Eng..

[66] Ebrahim A.A.M., El-Apasery M.A. (2023). A Facile Route for Removal of Reactive Dye Red 195 by Using Geopolymer Based on Bentonite. Egypt. J. Chem..

[67] Fitriani E., Purbasari A. (2021). Application of low-cost mesoporous geopolymer for dye waste removal. IOP Conference Series: Materials Science and Engineering.

[68] Al-Mashaqbeh A., El-Eswed B., Banat R., Khalili F.I. (2018). Immobilization of organic dyes in geopolymeric cementing material. Environ. Nanotechnol. Monit. Manag..

[69] Luhar I., Luhar S., Abdullah M.M.A.B., Razak R.A., Vizureanu P., Sandu A.V., Matasaru P.-D. (2021). A State-of-the-Art Review on Innovative Geopolymer Composites Designed for Water and Wastewater Treatment. Materials.

[70] He R., Dai N., Wang Z. (2020). Thermal and Mechanical Properties of Geopolymers Exposed to High Temperature: A Literature Review. Adv. Civ. Eng..

[71] Azimi E.A., Al Bakri Abdullah M.M., Ming L., Yong H.C., Hussin K., Aziz I.H. (2016). Processing, and properties of geopolymers as thermal insulating materials: A review. Rev. Adv. Mater. Sci..

[72] Lahoti M., Tan K.H., Yang E.-H. (2019). A critical review of geopolymer properties for structural fire-resistance applications. Constr. Build. Mater..

[73] Galiano Y.L., Leiva C., Arenas C., Arroyo F., Vilches L., Pereira C.F., Villegas R. (2017). Behavior of Fly Ash-Based Geopolymer Panels Under Fire. Waste Biomass.

[74] (1995). Leaching Characteristics of Solid Earthy and Stony Building and Waste Materials—Leaching Tests—Determination of the Leaching of Inorganic Components from Buildings and Monolitic Waste Materials with the Diffusion Test.

[75] Zhang H.Y., Kodur V., Qi S.L., Cao L., Wu B. (2014). Development of metakaolin–fly ash based geopolymers for fire resistance applications. Constr. Build. Mater..

[76] Zhang H., Kodur V., Cao L., Qi S. (2014). Fiber Reinforced Geopolymers for Fire Resistance Applications. Procedia Eng..

[77] Alzeebaree R., Mawlod A.O., Amen D.K., Younis K.H., Mohammedameen A. (2021). Fire Resistance Performance of Fiber Reinforced Geopolymer Concrete: Review. E3S Web of Conferences.

[78] Luhar S., Nicolaides D., Luhar I. (2021). Fire Resistance Behaviour of Geopolymer Concrete: An Overview. Buildings.

[79] Li H., Peng X., Li J., Li L., Hu D., Xiang Y., Han L., Xu Z. (2022). Preparation and fireproofing performance of the wollastonite-metakaolin-based geopolymer foams. Mater. Lett..

[80] Sakkas K., Kapelari S., Panias D., Nomikos P.P., Sofianos A. (2014). Fire Resistant K-Based Metakaolin Geopolymer for Passive Fire Protection of Concrete Tunnel Linings. Open Access Libr. J..

[81] Abbass A.M., Firdous R., Yankwa Djobo J.N.Y., Stephan D., Elrahman M.A. (2023). The role of chemistry and fineness of metakaolin on the fresh properties and heat resistance of blended fly ash-based geopolymer. SN Appl. Sci..

[82] Le V.S., Louda P., Tran H.N., Nguyen P.D., Bakalova T., Ewa Buczkowska K., Dufkova I. (2020). Study on Temperature-Dependent Properties and Fire Resistance of Metakaolin-Based Geopolymer Foams. Polymers.

[83] Aziz I.H., Abdullah M.M.A.B., Yong H.C., Ming L.Y., Hussin K., Kadir A.A., Azimi E.A. (2016). Manufacturing of Fire Resistance Geopolymer: A Review. MATEC Web of Conferences.

[84] Hassan A., Arif M., Shariq M., Alomayri T., Pereira S. (2023). Fire resistance characteristics of geopolymer concrete for environmental sustainability: A review of thermal, mechanical and microstructure properties. Environ. Dev. Sustain..

[85] Islam S., Bhat G. (2019). Environmentally-friendly thermal and acoustic insulation materials from recycled textiles. J. Environ. Manag..

[86] Zhang Z., Provis J.L., Reid A., Wang H. (2015). Mechanical, thermal insulation, thermal resistance, and acoustic absorption properties of geopolymer foam concrete. Cem. Concr. Compos..

[87] Soe P.S., Sornlar W., Wannagon A., Chaysuwan D. (2023). Mechanical and thermal properties of bottom ash-based porous geopolymer as thermal insulation material for construction. J. Mater. Cycles Waste Manag..

[88] Ahmed M.M., El-Naggar K.A.M., Tarek D., Ragab A., Sameh H., Zeyad A.M., Tayeh B.A., Maafa I.M., Yousef A. (2021). Fabrication of thermal insulation geopolymer bricks using ferrosilicon slag and alumina waste. Case Stud. Constr. Mater..

[89] Bai C., Franchin G., Elsayed H., Zaggia A., Conte L., Li H., Colombo P. (2017). High-porosity geopolymer foams with tailored porosity for thermal insulation and wastewater treatment. J. Mater. Res..

[90] Ekiz Bariş K., Tanaçan L. (2022). Natural pozzolan–based green geopolymer foam for thermal insulation. J. Sustain. Constr. Mater. Technol..

[91] Łach M., Korniejenko K., Mikuła J. (2016). Thermal insulation and thermally resistant materials made of geopolymer foams. Procedia Eng..

[92] Shahedan N.F., Al Bakri Abdullah M.M., Mahmed N., Ming L.Y., Abd Rahim S.Z., Aziz I.H.A., Kadir A.A., Sandu A.V., Ghazali M.F. (2022). Thermal Insulation and Mechanical Properties in the Presence of Glass Bubble in Fly Ash Geopolymer Paste. Arch. Metall. Mater..

[93] Rashad A.M. (2019). Insulating and fire-resistant behaviour of metakaolin and fly ash geopolymer mortars. Constr. Mater..

[94] Fang Y., Ahmad M.R., Lao J.C., Qian L.P., Dai J.G. (2023). Development of artificial geopolymer aggregates with thermal energy storage capacity. Cem. Concr. Compos..

[95] Zhang H., Gao H., Bernardo E., Lei S., Wang L. (2023). Thermal energy storage performance of hierarchical porous kaolinite geopolymer based shape-stabilized composite phase change materials. Ceram. Int..

[96] Jacob R., Trout N., Solé A., Clarke S., Fernández A.I., Cabeza L.F., Saman W., Bruno F. (2020). Novel geopolymer for use as a sensible storage option in high temperature thermal energy storage systems. AIP Conference Proceedings.

[97] Jacob R., Trout N., Raud R., Clarke S., Steinberg T.A., Saman W., Bruno F. (2016). Geopolymer encapsulation of a chloride salt phase change material for high temperature thermal energy storage. AIP Conference Proceedings.

[98] Yang J.L., Zhang W.B., Chai S.S., Theint M.M., Yin Y., Yang Z.Q., Li J.J., Yi Y.H., Ma X.J. (2023). A geopolymer membrane for application in a structural mechanics and energy storage difunctional supercapacitor. Phys. Chem. Chem. Phys..

[99] Rahjoo M., Goracci G., Gaitero J.J., Martauz P., Rojas E., Dolado J.S. (2022). Thermal Energy Storage (TES) Prototype Based on Geopolymer Concrete for High-Temperature Applications. Materials.

[100] Rahjoo M., Goracci G., Martauz P., Rojas E., Dolado J.S. (2022). Geopolymer Concrete Performance Study for High-Temperature Thermal Energy Storage (TES) Applications. Sustainability.

[101] Zhang L., Wang Y., Ding B., Gu J., Cai J. (2023). Study on the mechanical and thermal properties of one-part geopolymer composite for high performance in energy piles. Case Stud. Constr. Mater..

[102] Díaz E.E.S., Barrios V.A.E. (2022). Development, and use of geopolymers for energy conversion: An overview. Constr. Build. Mater..

[103] Freire A.L., José H.J., Moreira R.F.P.M. (2022). Potential applications for geopolymers in carbon capture and storage. Int. J. Greenh. Gas Control.

[104] Sarkar M., Maiti M., Maiti S., Xu S., Li Q. (2018). ZnO-SiO_2_ nanohybrid decorated sustainable geopolymer retaining anti-biodeterioration activity with improved durability. Mater. Sci. Eng. C.

[105] Kang X., Ye H. (2023). Structural composition of antibacterial zinc-doped geopolymers. Dalton Trans..

[106] Rondinella A., Furlani E., Dell’Antone L., Marin E., Boschetto F., Sordetti F., Lanzutti A., Andreatta F., Fedrizzi L., Maschio S. (2022). Mechanical and antibacterial behavior of multilayered geopolymer coatings on Ti6Al4V alloys. J. Mater. Sci..

[107] Hashimoto S., Machino T., Takeda H., Daiko Y., Honda S., Iwamoto Y. (2015). Antimicrobial activity of geopolymers ion-exchanged with copper ions. Ceram. Int..

[108] Růžek V., Novosád J., Buczkowska K.E. (2023). Geopolymer Antimicrobial and Hydrophobic Modifications: A Review. Ceramics.

[109] Armayani M., Pratama M.A. (2017). The Properties of Nano Silver (Ag)-Geopolymer as Antibacterial Composite for Functional Surface Materials. MATEC Web of Conferences.

[110] Lira B.C.S., Dellosa S.B.A., Toh C.I.L., Quintero A.P.A., Nidoy A.L.S., Cerna K.D., Yu D.E.C., Janairo J.I.B., Promentilla M.A.B. (2019). Coal Fly Ash-based Geopolymer Spheres Coated with Amoxicillin and Nanosilver for Potential Antibacterial Applications. ASEAN J. Chem. Eng..

[111] Cerna K.D., Janairo J.I., Promentilla M.A. (2019). Development of nanosilver-coated geopolymer beads (AgGP) from fly ash and baluko shells for antimicrobial applications. MATEC Web of Conferences.

[112] Gutiérrez R.M.-d., Villaquirán-Caicedo M., Ramírez-Benavides S., Astudillo M., Mejía D. (2020). Evaluation of the Antibacterial Activity of a Geopolymer Mortar Based on Metakaolin Supplemented with TiO_2_ and CuO Particles Using Glass Waste as Fine Aggregate. Coatings.

[113] Zhang Z., Yao X., Zhu H. (2010). Potential application of geopolymers as protection coatings for marine concrete: I. Basic properties. Appl. Clay Sci..

[114] Jiang C., Wang A., Bao X., Ni T., Ling J. (2020). A review on geopolymer in potential coating application: Materials, preparation and basic properties. J. Build. Eng..

[115] Aguirre-Guerrero A.M., Robayo-Salazar R.A., de Gutiérrez R.M. (2017). A novel geopolymer application: Coatings to protect reinforced concrete against corrosion. Appl. Clay Sci..

[116] Zhang Z., Yao X., Zhu H. (2010). Potential application of geopolymers as protection coatings for marine concrete: II. Microstructure and anticorrosion mechanism. Appl. Clay Sci..

[117] Yang N., Das C.S., Xue X., Li W., Dai J.-G. (2022). Geopolymer coating modified with reduced graphene oxide for improving steel corrosion resistance. Constr. Build. Mater..

[118] Tomar A.S., Gupta R., Singh A., Salammal S.T., Khan M.A., Mishra D. (2022). Evaluation of corrosion protective properties of fly ash-red mud based geopolymer coating material for mild steel. Mater. Today Proc..

[119] Gupta R., Tomar A.S., Mishra D., Sanghi S.K. (2021). Multifaceted geopolymer coating: Material development, characterization, and study of long term anti-corrosive properties. Microporous Mesoporous Mater..

[120] Tomar A.S., Gupta R., Bijanu A., Tanwar D., Singh A., Salammal S.T., Dhand C., Mishra D. (2023). TiO_2_- geopolymer based novel corrosion protective micro-coatings to emaciate mild steel oxidation in severe environments. Constr. Build. Mater..

[121] Zainal F.F., Fazill M.F., Kamarudin H., Rahmat A., Al Bakri Abdullah M.M., Wazien W. (2018). Effect of Geopolymer Coating on Mild Steel. Solid State Phenom..

[122] Morla P., Gupta R., Azarsa P., Sharma A. (2021). Corrosion Evaluation of Geopolymer Concrete Made with Fly Ash and Bottom Ash. Sustainability.

[123] Omer L.M., Gomaa M.S., Sufe W.H., Elsayed A.A., Elghazaly H.A. (2022). Enhancing corrosion resistance of RC pipes using geopolymer mixes when subjected to aggressive environment. J. Eng. Appl. Sci..

[124] Sobhan K., Martinez F.J., Reddy D.V. (2021). Corrosion resistance of fiber-reinforced geopolymer structural concrete in a simulated marine environment. Can. J. Civ. Eng..

